# Diffuse parenchymal lung disease as first clinical manifestation of GATA-2 deficiency in childhood

**DOI:** 10.1186/s12890-015-0006-2

**Published:** 2015-02-10

**Authors:** Tamara Svobodova, Ester Mejstrikova, Ulrich Salzer, Martina Sukova, Petr Hubacek, Radoslav Matej, Martina Vasakova, Ludmila Hornofova, Marcela Dvorakova, Eva Fronkova, Felix Votava, Tomas Freiberger, Petr Pohunek, Jan Stary, Ales Janda

**Affiliations:** Department of Pediatrics, 2nd Faculty of Medicine, Charles University in Prague and University Hospital Motol, Prague, Czech Republic; Department of Pediatric Hematology and Oncology, 2nd Faculty of Medicine, Charles University in Prague and University Hospital Motol, Prague, Czech Republic; Center for Chronic Immunodeficiency (CCI), University Medical Center and University of Freiburg, Freiburg im Breisgau, Germany; Department of Medical Microbiology, 2nd Faculty of Medicine, Charles University in Prague and University Hospital Motol, Prague, Czech Republic; Department of Pathology and Molecular Medicine, Thomayer’s University Hospital, Prague, Czech Republic; Department of Respiratory Medicine, Thomayer’s University Hospital, Prague, Czech Republic; Department of Pathology and Molecular Medicine, 2nd Faculty of Medicine, Charles University in Prague and University Hospital Motol, Prague, Czech Republic; Department of Radiology, 2nd Faculty of Medicine, Charles University in Prague and University Hospital Motol, Prague, Czech Republic; Department of Pediatrics, 3rd Faculty of Medicine, Charles University in Prague and University Hospital Kralovske Vinohrady, Prague, Czech Republic; Molecular Genetics Lab, Centre for Cardiovascular Surgery and Transplantation, Brno, Czech Republic; Department of Clinical Immunology and Allergology, Medical Faculty, Masaryk University Brno, Brno, Czech Republic; Department of Pediatric Infectious Diseases and Rheumatology, Center of Pediatrics and Adolescent Medicine, University Medical Center and University of Freiburg, Mathildenstrasse 1, 79106 Freiburg im Breisgau, Germany

**Keywords:** Primary immunodeficiency, GATA-2 deficiency, Diffuse parenchymal lung disease, EBV Viremia, Childhood

## Abstract

**Background:**

GATA-2 transcription factor deficiency has recently been described in patients with a propensity towards myeloid malignancy associated with other highly variable phenotypic features: chronic leukocytopenias (dendritic cell-, monocyto-, granulocyto-, lymphocytopenia), increased susceptibility to infections, lymphatic vasculature abnormalities, and sensorineural deafness. Patients often suffer from opportunistic respiratory infections; chronic pulmonary changes have been found in advanced disease.

**Case presentation:**

We present a case of a 17-year-old previously healthy Caucasian male who was admitted to the hospital with fever, malaise, headache, cough and dyspnea. A chest X-ray revealed bilateral interstitial infiltrates and pneumonia was diagnosed. Despite prompt clinical improvement under antibiotic therapy, interstitial changes remained stable. A high resolution computer tomography showed severe diffuse parenchymal lung disease, while the patient’s pulmonary function tests were normal and he was asymptomatic. Lung tissue biopsy revealed chronic reparative and resorptive reaction with organizing vasculitis. At the time of the initial presentation to the hospital, serological signs of acute infection with Epstein-Barr virus (EBV) were present; EBV viremia with atypical serological response persisted during two-year follow up. No other infectious agents were found. Marked monocytopenia combined with B-cell lymphopenia led to a suspicion of GATA-2 deficiency. Diagnosis was confirmed by detection of the previously published heterozygous mutation in *GATA2* (c.1081 C > T, p.R361C). The patient’s brother and father were both carriers of the same genetic defect. The brother had no clinically relevant ailments despite leukocyte changes similar to the index patient. The father suffered from spondylarthritis, and apart from B-cell lymphopenia, no other changes within the leukocyte pool were seen.

**Conclusion:**

We conclude that a diagnosis of GATA-2 deficiency should be considered in all patients with diffuse parenchymal lung disease presenting together with leukocytopenia, namely monocyto-, dendritic cell- and B-lymphopenia, irrespective of severity of the clinical phenotype. Genetic counseling and screening for *GATA2* mutations within the patient’s family should be provided as the phenotype is highly variable and carriers without apparent immunodeficiency are still in danger of developing myeloid malignancy. A prompt recognition of this rare condition helps to direct clinical treatment strategies and follow-up procedures.

## Background

Defects of transcription factor GATA-2 have recently been identified in a few overlapping phenotypes associated with myeloid malignancies: dendritic cell, monocyte, B- and NK-cell deficiency; MonoMAC syndrome (monocytopenia with *Mycobacterium avium* complex infections); Emberger syndrome (early onset primary lymphedema, multiple warts, sensorineural deafness, dysmorphism); and familial MDS/AML with no additional known phenotype. These syndromes share autosomal-dominant inheritance with variable manifestation of immunodeficiency [1-9]. The respiratory tract is frequently affected by viral, fungal or mycobacterial infections. Chronic lung tissue changes and pulmonary alveolar proteinosis (PAP), as well as pulmonary arterial hypertension, have been described in adult patients [1,7-9].

We present an adolescent male with GATA-2 deficiency and early manifestation of diffuse parenchymal lung disease (DPLD) as well as an atypical course of Epstein-Barr virus (EBV) infection.

## Case presentation

**Figure 1:**
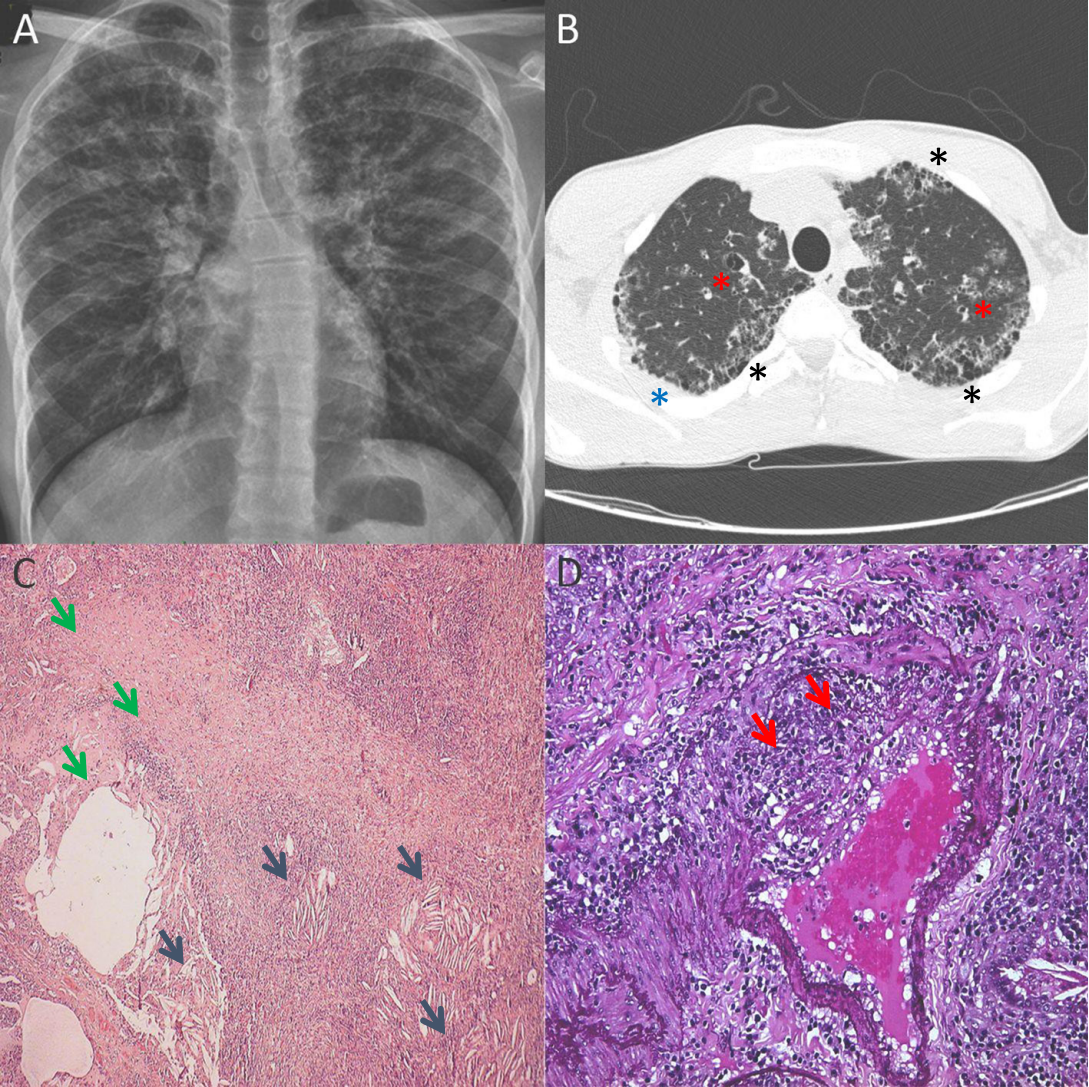
**Pulmonary changes in the index patient. A**: Diffuse bilateral linear and reticular opacities, compatible with interstitial pulmonary involvement (chest X-ray). **B**: Diffuse subpleural fibrotic changes – honeycomb (black asterisk), areas of subpleural consolidations (blue asterisk) and bronchectasis (red asterisk) in the upper lobes (high-resolution computer tomography scan). **C**: Chronic reparative and resorptive reaction: fibrosis and cystic rearrangement (green arrows) and cholesterol clefts (blue arrows) in the upper left lobe (hematoxylin and eosin tissue stain; original magnification 50x). **D**: Thickened arterial wall, destruction of the elastic layer, thrombosis showing organizing vasculitis (red arrows) in the upper left lobe (elastin tissue stain; original magnification 250x).

A 17-year-old Caucasian male presented to the hospital with acute fever, malaise, headache, cough and dyspnea. A bilateral pneumonia with signs of systemic inflammation corresponding to bacterial infection (C-reactive protein 210 mg/l) was diagnosed and antibiotic treatment initiated. No causative microorganism was identified. Despite rapid clinical improvement, chest X-ray showed persistent interstitial changes (Figure 1A). A subsequent high-resolution computer tomography (HRCT) revealed marked lung damage suggestive of bronchiectasis with peribronchitis, fibrotisation, subpleural cystic remodeling (honey-combing) and emphysema (Figure 1B). Interestingly, pulmonary function tests showed normal vital capacity, total lung capacity as well as diffusing capacity (Table 1). Thus, we detected chronic lung changes with no functional correlate during the first episode of pneumonia in a previously healthy boy. Further investigations to unfold the cause for the diffuse parenchymal lung disease were initiated.

**Table 1:** **Results of pulmonary function tests**

	**Initial investigation**	**After 2-year follow-up**
FVC	4.58 l (85%)	4.82 l (81%)
FEV1	4.48 l/min (101%)	4.45 l/min (93%)
MEF50	7.32 l/min (140%)	7.17 l/min (121%)
TLC	6.14 l (92%)	6.09 l (78%)
DLCO	87%	81%

Legend: The first examination was performed two weeks after disease manifestation, there were more than 10 tests performed in the follow-up, all showing similar values. The numbers are related to normal values (parenthesis); the reference cohort has changed during the follow-up (< 18 and > 18 years of age). The shape of expiratory/inspiratory flow curve suggested intermittently mild periphery obstruction. *Abbreviations:* *FVC* Forced vital capacity, *FEV1* Forced expiratory volume in 1 second, *MEF* Maximal mid-expiratory flow, *TLC* Total lung capacity, *DLCO* Diffusing lung capacity.

A complete blood count showed leukocytopenia with marked monocytopenia (Table 2). Immunological assays detected B-cell lymphopenia with predominance of memory B cells. Despite the very low numbers of B cells, normal serum immunoglobulin levels of IgM and IgA and increased levels of IgG were present (23.6 g/l). Antibody response to routine vaccination was normal. No serum autoantibodies were found. Functional testing of granulocytes (respiratory burst test: analysis of the ability of granulocytes to release reactive oxygen species after in vitro stimulation) and of T cells (evaluation of proliferative response of T cells to various in vitro stimuli) excluded chronic granulomatous disease and T-cell proliferation defects.

**Table 2:** **Leukocyte and lymphocyte subsets**

	**Initial investigation**	**After 2-year follow-up**	**Normal values**
	**× 10^9/l (%)**	**× 10^9/l (%)**	**× 10^9/l (%)**
Leukocytes	2.8	5.4	4.0 - 10.0
Monocytes	0.014 (0.5*)	0.028 (0.5*)	0.12 - 1.0 (3 - 10*)
Lymphocytes	0.773 (27.6*)	2.120 (39.3*)	1.0 - 3.3 (25 - 33*)
T cells (CD3+)	0.659 (85.3**)	1.696 (80.0**)	2.0 - 6.5 (55 - 88**)
B cells (CD19+)	0.015 (1.9**)	0.042 (2.0**)	0.4 - 3.3 (11 - 45**)
NK cells (CD56 + 16+)	0.085 (11.0**)	0.382 (18.0**)	0.1 - 1.0 (6 - 26**)
CD4/8 index	1.3	1.7	1 - 3

Legend: The initial leuko- and lymphopenia resolved; the profound monocyto- and B-cell lymphopenia persisted. The B cells were predominantly of memory phenotype. Myeloid as well as plasmacytoid dendritic cells were missing. In the parenthesis percentage of cells out of (*) leukocytes and (**) lymphocytes, respectively, are shown.

Serology corresponded with primary EBV infection (Table 3). However, the EBV viral load in peripheral blood was low. Bronchoalveolar lavage (BAL) showed EBV presence in the bronchial fluid. Immunological analysis of the BAL fluid showed lymphocytosis with predominance of CD8^pos^ with increased HLA-DR expression (especially on CD3^pos^8^pos^); alveolar macrophages were present, CD1a^pos^ cells were not detected. No PAS (Periodic Acid–Schiff) positive material was evident in the alveolar macrophages (Table 4).

**Table 3:** **Serologic and molecular genetic analysis of Epstein-Barr virus infection**

		**Initial investigation**	**After 2-year follow-up**
Anti-VCA IgG	U/ml	63	>750
Anti-VCA IgM	U/ml	>160	47
Anti-EA-D IgG	U/ml	22	>150
Anti-EBNA 1 IgG	U/ml	Negative	Negative
Viral load in PB	Copies/10000 g.e	288	31
Viral load in BAL	Copies/ml	22000	6000

*Abbreviations:* VCA viral capsid antigen, *EA-D* early antigen component, *EBNA 1* Epstein-Barr virus nuclear antigen 1, *PB* peripheral blood, *BAL* bronchoalveolar fluid, *g.e.* genomic equivalent.

**Table 4:** **Cytology analysis of bronchoalveolar fluid from the initial bronchoscopy investigation**

Lymphocytes	60.4%	of leukocytes
CD3 + DR+	35%	of CD3+ cells
CD4/8 ratio	0.6	
Neutrophile granulocytes	6.8%	of leukocytes
Alveolar macrophages	31.6%	of leukocytes
Plasmatic cells	1.2%	of leukocytes

Neither bacterial, fungal, mycobacterial (including non-tuberculous mycobacteria) nor viral (cytomegalovirus, human herpes virus 6, varicella zoster virus, human herpes virus, respiratory syntitial virus, influenza, adenovirus, enterovirus, coronavirus, parainfluenza, human rhinovirus, human metapneumovirus, bokavirus and papillomavirus tested) infection was revealed via culture, serology or molecular genetic testing in peripheral blood and bronchoalveolar fluid. Hence, the extensive microbiological analysis revealed only the presence of EBV in peripheral blood and lungs.

Histopathological investigation of the lung parenchyma was prompted. Thoracoscopic lung biopsy from a severely affected region of the right upper lobe showed fibrosis, cystic rearrangement and cholesterol clefts with signs of organizing pneumonia and vasculitis (Figure 1 C, D). Inflammatory infiltration was predominantly lymphoplasmocytic with presence of activated macrophages. No changes compatible with pulmonary alveolar proteinosis or other alveolar filling disorder were seen. Despite an EBV presence (2300 copies/10.000 genomic equivalents, g.e.) in the lung tissue found with polymerase chain reaction, hybridization probes for EBV-encoded small RNA (EBER) were negative in the histology slides. Thus, no clear relationship between EBV and the histopathological parenchymal changes could be stated.

Given the severe affliction of the lung parenchyma with fibrotic remodeling, ongoing inflammation with activated CD8^pos^ T cells in the bronchoalveolar fluid and lack of clear evidence for an infectious cause, a treatment with an oral steroid was initiated to suppress further tissue destruction. A prophylactic antibiotic (azithromycin) was added and the patient was closely monitored. Immunoglobulin levels normalized and signs of systemic inflammation regressed. After 6 months a stable finding was documented via HRCT and no clinical symptoms were present. The EBV viral load remained low in peripheral blood. Minimal presence of the virus was seen in repeated BAL. However, the serological signs of active EBV infection persisted and no EBNA antibodies were detected at the follow up. No lymphoproliferation was present and the patient remained asymptomatic. The condition was classified as persistent EBV viremia accompanied by an atypical serological response. Molecular genetic testing of *SH2D1A* was carried out. A normal result excluded X-linked lymphoproliferative disease, the most common inborn cause of abnormal immunological reaction to EBV infection. Details on other possible genetic causes, not yet tested in our patient, are in the Discussion.

The patient has a history of occasional uncomplicated respiratory infections; At the age of 13 years he suffered from acute bronchitis, a chest X-ray was performed and retrospective analysis of the image showed some interstitial changes present already at that time. Monocytopenia was documented as early as at the age of 10 years.

The persistent profound monocytopenia and B- lymphocytopenia at follow up prompted *GATA2* sequencing. The diagnosis of GATA-2 deficiency was confirmed by the finding of a known heterozygous pathogenic variation c.1081 C > T (p.R361C) [10]. Myeloid malignancy was excluded by morphological, flow cytometric and cytogenetic analysis of the bone marrow aspirate. Detailed immunophenotypic analysis of the bone marrow showed suppression of CD34^pos^ and CD117^pos^ precursors; impairment of B-cell lineage (only 1.4% B cells were present, out of those plasma cells constituted 42% and mature CD20^pos^CD10^neg^ cells 37%, the precursors CD34^pos^CD10^pos^ were scarce) and lower percentage of monocytes as well as their progenitors (CD14^high^CD45^pos^SSC^med^). Additional testing showed lack of myeloid and plasmacytoid dendritic cells.

The immunosuppressive treatment was stopped and the patient was further treated with prophylactic antibiotics and antimycotics. Vaccination against human papillomavirus (HPV) was performed as recommended [9]. He has been monitored closely, including regular checks of bone marrow aspirate for early detection of clonal myeloid proliferation. In case of myelodysplasia, transplantation of hematopoietic stem cells would be initiated. After two years of follow-up the patient did not develop any clinical symptoms. He was treated once for *Pseudomonas aeruginosa* found in the bronchoalveolar fluid detected in the second BAL analysis performed 6 months after the first one. Otherwise, there were no clinical signs of increased susceptibility to infection. Pulmonary function tests remained normal, no progression of the pulmonary parenchyma affliction have been detected so far (Tables 1, 2 and 3).

The same heterozygous mutation in *GATA2* was found in the patient’s 13-year-old brother and 45 year-old father, whereas his mother was healthy. The brother had been without any clinical symptoms so far, blood tests revealed leukocytopenia and marked monocytopenia. HRCT scan showed normal parenchyma, no EBV activity was documented. The father suffered from bilateral ankylosing spondylitis (HLA-B27 positive). Apart from low B-cell numbers (2.4% CD19^pos^ cells of lymphocytes, with prevailing memory phenotype: 70% CD27^pos^ cells out of B cells; Norm < 47%) no leukocyte count abnormalities were detected.

The lymphocyte changes in the three family members carrying the *GATA2* mutation stimulated investigation of bone marrow output. Newly emerging T and B cells can be assessed via T-cell recombination circle (TREC) and kappa-deleting element recombination circle (KREC) analysis in the peripheral blood [11,12]. As expected, both siblings had no detectable KREC copies in the peripheral blood, indicating severe impairment of B cell development. TREC analysis showed normal results. Bone marrow examination of the younger brother also showed complete negativity of KREC with normal TREC copies. KREC copies were absent in the peripheral blood of the father as well. The KREC/TREC copies were normal in the unaffected mother.

Interestingly, DNA obtained from the newborn Guthrie card of the younger brother was analyzed showing a normal amount of KREC/TREC copies. This indicates that the impairment in B lymphocyte development occurred postnatally.

## Discussion

GATA-2 deficiency is a protean disease with a broad spectrum of symptoms. Most of the patients present with hematological abnormalities (cytopenias, early-onset myeloid malignancies) and an increased susceptibility to opportunistic infections [1-9]. In the recently published cohort of 57 patients treated at the National Institute of Health (NIH, Bethesda, USA) [9] 70% of the patients had severe viral infections, particularly infection with HPV (63%) presenting with recalcitrant warts, condylomata, and/or dysplasia. Severe herpesvirus infections were present in 35% of patients: recurrent herpes stomatitis, esophagitis, genital infection, severe varicella in 11% of cases, and cytomegalovirus pneumonia or disseminated disease. Interestingly, in 11% of patients persistent EBV viremia similar to our patient was documented; in 2 patients EBV-positive skin tumors occurred. Infection with non-tuberculous mycobacteria was seen in 53%, severe bacterial infection was observed in 49% and severe invasive fungal infection in 16% of the patients. Eighteen percent of patients showed no increased susceptibility to infection. Additionally, vascular/lymphatic defects (venous thrombosis, lymphedema), sensorineural hearing loss, miscarriages and hypothyroidism were found [1-9].

Pulmonary involvement in GATA-2 deficiency is frequent, involving infections and PAP [1,3,8,9], particularly in more advanced stages of the disease. In the NIH cohort, 79% and 63% of the patients had diffusion and ventilatory defects, respectively. PAP was found in 18% and pulmonary arterial hypertension in 9% of patients. Structural abnormalities included nodules, reticular and ground glass opacities, subpleural blebbing, “crazy paving”, and paraseptal emphysema [9]. Similar picture could be seen in our patient. The surprisingly normal pulmonary function test results in our patient could possibly be explained by localized affliction of the pulmonary tissue. The infiltrated and fibrotic tissue decreased the elasticity of the lung parenchyma, however, there was still enough normal tissue that kept the static volumes and transfer factor normal (Table 1). Unfortunately, it is not possible to compare our findings with other pediatric patients as the data on pulmonary infliction in children are scarce. There were 24 children in the NIH cohort. Data on the pulmonary function tests were presented for only 6 of them (median age at testing 16 years, range 12–17 years; median time from disease manifestation 2.5 years, range 0–16 years). All those children suffered from myeloid malignancy, in four of them a chronic infection with herpesviruses or mycobacteria species was documented. Mild to severe diffusion defects were found in all tested patients, in two children a bronchial obstruction was seen. No information on structural lung changes in the affected children was provided [9].

The median age at initial presentation in the NIH cohort was 20 years but was highly variable (range 5 months – 78 years). Of note, four individuals (7%) had no apparent clinical manifestations as of the last follow-up (range 5–55 years). The proportion of patients without symptoms was 50% by age 20, 25% by age 30, and 16% by age 40 irrespective of the type of genetic change in *GATA2*. The phenotype varied within families significantly [9]. This fact strongly argues for a substantial impact of epigenetic, infectious and environmental factors on disease manifestation. Effects of germline or somatic mutations in other genes may play a role as well. This may explain the variability of symptoms in the three individuals carrying the same *GATA2* mutation within our index family.

An intriguing issue is the etiology of the chronic diffuse parenchymal lung tissue changes in our patient in the absence of respiratory symptoms. The extensive investigations revealed only EBV presence in peripheral blood as well as in pulmonary tissue without specific tissue changes or clinically apparent EBV infection (e.g. mononucleosis-like symptoms, lymphoproliferation). As the patient presented with serological signs of acute EBV infection whereas the pulmonary changes were chronic, a decisive role of EBV in the pathogenesis of the pulmonary tissue changes in our patient was improbable. Possibly repeated mild infections in an environment of impaired regulation of the endothelial nitric oxide synthetase expression [13], defective phagocytosis and impaired GM-CSF signaling in pulmonary macrophages [14,15] played a role in the pathogenesis of the chronic pulmonary inflammatory changes.

Poor control of EBV replication resulting in persistent EBV viremia irrespective of lung involvement is a known phenomenon in GATA-2 deficient patients [1,7-9]. It has been shown that the inability to confine viral infections in patients with GATA-2 deficiency correlates well with the extent of cytopenias, namely with the lack of DC-, NK- and CD4^pos^ T-cells. Similarly, the defective antibody response at more advanced stages is associated with B cell lymphopenia [8,9]. However, with regards to the possible oligogenic etiology of immunodeficiency in GATA-2 deficiency, impact of other genes implicated in EBV control should be considered. We have excluded only the most common syndrome – X-linked lymphoproliferative disease type 1 caused by a defect in an adapter protein SAP, involved in signalling of cell-cell interactions. Other molecules implicated in EBV control encompass for example: the ubiquitously expressed XIAP with both antiapoptotic function and multiple signalling pathway connections; the surface molecule CD27, important for intercellular communication; the NK cell activating receptor for antibody-dependent cell cytotoxicity (CD16), and minichromosome maintenance 4 (MCM4) crucial for NK-cell function; or IL-2-inducible T-cell kinase (ITK), coronin1A, serine-threonine kinase (STK)4 and magnesium transporter, MAGT1, indispensable for T-cell receptor signalling and T-cell homeostasis [16].

The search for a GATA-2 defect in our patient was prompted by the abnormalities in the leukocyte and lymphocyte counts. Another serum marker useful in diagnostics as well as in monitoring of the disease progression (correlating with cytopenia) is the stem cell growth Fms-related tyrosine kinase 3 ligand (Flt3 ligand) [8]. We have shown that the newborn screening using KREC/TREC analysis [17] cannot be used to screen for GATA-2 deficiency.

The prognosis of individuals with *GATA2* mutations is difficult to establish due to high clinical variability, incomplete penetrance and lack of close phenotype-genotype correlation data [8,9,18]. Antibiotics (e.g. azithromycin) and HPV vaccination are the recommended prophylactic measures [9]. Use of steroids or other immunosuppressive therapy is not indicated and exclusion of immunodeficiency in DPLD prior to use is warranted. A large proportion of patients will develop myeloid malignancy later in life [1,7-9]. The only curative therapy is allogeneic hematopoietic stem cell transplantation. Two patients with GATA-2 deficiency with pulmonary involvement transplanted for advanced MDS were reported to have profited significantly from this procedure [7].

## Conclusion

Diffuse parenchymal lung diseases are a heterogeneous group of disorders with an often insidious onset of symptoms [19]. The underlying immunodeficiency may not be apparent and an immunological and genetic work-up is required, in particular if abnormalities in peripheral leukocyte counts are revealed. As demonstrated in our patient, an aberrant immune response to common respiratory infections may result in diffuse lung disease with bronchial and bronchiolar damage, significant chronic changes of pulmonary parenchyma and fibrotic remodeling. The structural changes might be present prior to any severe infection.

Diffuse parenchymal lung disease may become the first manifestation of the GATA-2 deficiency. Early genetic diagnosis is critical to direct clinical management, prophylaxis, transplantation, and family screening.

## Consent

Written informed consent was obtained from the patient and the family for publication of this Case report and any accompanying images. A copy of written consent is available for review by the Editor of this journal.
